# Inadequacy of nutrients intake among pregnant women in the Deep South of Thailand

**DOI:** 10.1186/1471-2458-10-572

**Published:** 2010-09-24

**Authors:** Phnom Sukchan, Tippawan Liabsuetrakul, Virasakdi Chongsuvivatwong, Praneed Songwathana, Vosasit Sornsrivichai, Metta Kuning

**Affiliations:** 1 Development of Health Strategy Unit, Narathiwat Provincial Public Health Office, Narathiwat 96000, Thailand; 2 Epidemiology Unit, Faculty of Medicine, Prince of Songkla University, Hat Yai, Songkhla 90112, Thailand; 3 Nursing Faculty, Prince of Songkla University, Hat Yai, Songkhla 90112, Thailand; 4 Faculty of science, Prince of Songkla University, Pattani campus, Pattani 94000, Thailand

## Abstract

**Background:**

The deep south of Thailand is an area which has been affected by violence since 2004, yet the concurrent coverage of antenatal care has remained at over 90%. Our study aimed to describe the prevalence of nutrient inadequacy among pregnant women who attended antenatal care clinics in hospitals in the study area and assess factors associated with nutrient inadequacy.

**Methods:**

Pregnant women from four participating hospitals located in lower southern Thailand were surveyed during January-December 2008. Nutrient intake was estimated based on information provided by the women on the amount, type and frequency of various foods eaten. Logistic regression was used to assess individual and community factors associated with inadequate nutrient intake, defined as less than two thirds of the recommended dietary allowance (RDA).

**Results:**

The prevalence of carbohydrate, protein, fat, calories, calcium, phosphorus, iron, thiamine, riboflavin, retinol, niacin, vitamin C, folic acid and iodine inadequacy was 86.8%, 59.2%, 78.0%, 83.5%, 55.0%, 29.5%, 45.2%, 85.0%, 19.2%, 3.8%, 43.2%, 0.8%, 0.0% and 0.8%, respectively. Maternal age, education level, gestational age at enrolment and pre-pregnancy body mass index and level of violence in the district were significantly associated with inadequacy of carbohydrate, protein, phosphorus, iron, thiamine and niacin intake.

**Conclusions:**

Nutrient intake inadequacy among pregnant women was common in this area. Increasing levels of violence was associated with nutrient inadequacy in addition to individual factors.

## Background

Nutrient intake is important to the well-being of pregnant women and the fetus. Inadequate nutrient intake can lead to maternal anemia, increasing the risk for other maternal morbidities and mortality, fetal growth retardation and low fetal birth weight [1,2]. Inadequacy of macronutrient and micronutrient intake in pregnant women has been previously reported in developing countries such as Bangladesh[3], China [4], Sudan[5], Nigeria[6] and in developed countries such as the USA [7]. In Thailand, the national prevalence of iron deficiency anemia among pregnant women was 13% [8] but the prevalence in the southern region was as high as 37.8% [9]. In addition, 55% of women had their daily nutrient intake less than the minimum acceptable levels based on the Recommended Dietary Allowance (RDA) [10]. Inadequacy of micronutrient intake was also noted in a recent study in southern Thailand [11].

Previous studies have shown that family income, education and pregnancy body mass index (BMI) were associated with nutrient inadequacy [3,12,13]. In addition to these demographic and socioeconomic factors, occurrence of violence events stemming from social conflict can cause failure of food supplies and a worsening of health service systems [14,15]. An association between conflict situations within a community and malnutrition has been demonstrated in 21 countries in sub-Saharan Africa [16]. It is not unlikely then that a conflict situation in a community may influence nutrient inadequacy of the pregnant women who live there.

Narathiwat is located in the lower southern part of Thailand where the prevalence of anemia in pregnant women is higher than 20% and low birth weight is steadily reported at 9%. Importantly, the people living in this area have been besieged by violent conflicts since 2004. However, the local health systems are well-functioning and still being utilized. The proportion of live births delivered in institutions has risen from 78% in 2004 to 86% in 2008. Similarly, the percentage of women having their first antenatal visit before 12 weeks of gestation was 72% in 2004 and this increased to 85% in 2008. Due to the high coverage of early antenatal visits, there is a good opportunity to investigate the inadequacy of nutrient intake among these women with respect to both the magnitude of the problem and its associated factors. This information will be useful for further planning of interventions. Our study thus aimed to describe the daily dietary macronutrient and micronutrient intake regarding the six Thai food groups among pregnant women at the first half of pregnancy and to assess the prevalence of inadequacy of these nutrients and associated factors on individual and community characteristics.

## Methods

### Study design

A hospital-based cross-sectional survey was approved by the Ethics Committee of the Faculty of Medicine, Prince of Songkla University, Songkhla, Thailand (reference no. EC 50/354-012), the Ethics Committee of the Ministry of Public Health (reference no. 9/2008) and the Chief Medical Officers of Narathiwat Provincial Health Offices and the Directors of participating hospitals. All eligible pregnant women were invited to join the study. Written informed consent was obtained from all recruited subjects.

### Study setting

Narathiwat province was purposively selected to be the study setting because the prevalence of maternal anemia and low birth weight are high. In addition, the people living in this area are culturally different from those of people in other regions of the country. Hospitals from four districts, namely Ra-Ngae, Tak Bai, Su-Ngai Padi and Cho-Irong were chosen as the study hospitals. All pregnant women with a gestational age of 12 to 20 weeks, who had lived in Narathiwat province for at least one year prior to the study and had attended their first antenatal visit in one of the study hospitals during January-December 2008, were included in the study. High-risk pregnancies such as severe anemia of less than seven gm/dl, heart disease, AIDS, or any mental problems were criteria for exclusion.

### Sample size

The prevalence of inadequacy of nutrient intake in a previous study varied considerably [10], so the average prevalence (55%) was considered in this study. In order to estimate this prevalence with 95% confidence and a precision of 5%, a total of 380 pregnant women were needed.

### Data collection

A one-day training workshop was arranged by the investigators to allow the relevant staff of each participating hospital to be acquainted with the study details and data collection procedures. One full-time female research assistant was employed at each hospital to collect the data under regular supervision of the first author.

After receiving a full explanation on the purpose of the study, the women were given a self-administered structured questionnaire to ascertain their individual baseline characteristics. The table of food intake recommended for pregnant women published in an antenatal care handbook provided by the Ministry of Public Health, Thailand was used for guidance of food items required. The six food groups including rice, vegetable, meat, fruit, fat and milk were listed. The units for measuring the amount of each food was recorded as ladles for rice and vegetables, portions for fruit, tablespoons for meat, tea-spoons for fat and glasses for milk. Photographs of the types and amounts of foods were shown to the women to improve estimation of portion size. The subjects were requested to recall their average daily intake for breakfast, lunch, dinner and supper over the previous two weeks for all food groups and the amount eaten using the standard units mentioned above. If the consumed food was not in the list, then that kind of food was converted into an equivalent amount of the same food type. For example, noodles were converted into rice equivalent. Total food intake regarding types, amount and frequency were calculated into weight of macronutrient and micronutrient using the modified Food Nutrient Conversion Table endorsed by the Nutrition Division of the Ministry of Public Health, Thailand [17].

### Nutrient Variables

The two main outcome variables were level of macronutrients, which included carbohydrate, protein, fat and calories, and level of micronutrients, which included minerals (calcium, phosphorus and iron), vitamins (thiamine, riboflavin, retinol, niacin, vitamin c, folic acid) and iodine. Inadequacy of macronutrients and micronutrients was defined as having total nutrient intake lower than two-thirds of the RDA.

### Independent Variables

Individual characteristics obtained from each subject were age, religion, gestational age, occupation, education level, family income, number of family members, pre-pregnancy weight, height and number of live children. Community characteristics in 2008 on population density, average household income and literacy rate were obtained, while number of violent events, number of deaths and number of injuries in 2008 were retrieved from the Violence-related Injury Surveillance database [18]. The level of violence in study communities was determined by a weighted violence score which was retrieved from three parameters of violence, namely the number of violent events, number of injuries and number of deaths from violent events. These three parameters were divided by the mid-interval population to obtain the standardized scores of numbers of events, injured cases and dead cases, respectively. Factor analysis was then run on these three violence-related variables to obtain a weighted violence score for each district, reflecting the level of violence. Women living in the same district thus had the same weighted violence score.

### Statistical analysis

The data were computerized using Epidata 3.1 and analyzed using R software version 2.9.1 (The R Foundation for Statistical Computing 2008, Austria). The total daily intake of macronutrients and micronutrients was described using means and quartiles. Percentage of RDA (the actual quantities of nutrients divided by the RDA levels multiplied by 100) and percentage of inadequacy were computed. Logistic regression models using backward stepwise selection were fit to the data to estimate the effect of individual and community level factors on nutrient intake. The results were shown as odds ratios with 95% confidence intervals (95% CI). Statistical significance was considered for p values less than 0.05.

## Results

### District Characteristics

Table 1 shows the community characteristics in each study district in 2008. Ra-Ngae had the highest population while Chi-Irong had the highest population density. Ra-Ngae district had the highest average household income and violence rate compared to the other districts. The districts with a high violence rate (per 100,000 population) also had high injury and death rates. The most common violent events were bomb blasts and shootings.

**Table 1:** Community characteristics in 2008

	Districts			
	
Characteristics	Ra-Ngae	Tak Bai	Su-Ngai Padi	Cho-Irong
Area (km^2^)^a^	435.6	253.4	372.6	162.7
Population (persons)^a^	69754	48282	44453	36662
Population density^a^	160.1	191.5	119.3	225.3
Average household				
income (baht per year)^b^	75113.6	35543.8	37949.4	37218.8
Literacy rate (%)^b^	98.6	97.6	97.8	96.7
Number of violent events^c^	37	10	8	15
Violence rate^d^	53.0	20.7	17.9	40.9
Number of deaths^c^	31	2	3	8
Death rate^d^	44.4	4.1	6.7	21.8
Number of injuries^c^	64	24	9	47
Injury rate^d^	91.7	49.7	24.2	128.2
Case fatality rate (%)^c^	48.4	8.3	33.3	17.0

Source: ^a^Administration Department, Ministry of Interior, Thailand

^b^Narathiwat Social Development and Human Security Office

^c^Violence-related Injury Surveillence (VIS) database

^d^Per 100,000 persons

### Participant characteristics

Of 462 eligible women approached, 400 (86.5%) consented to participate in the study. Individual characteristics of these participants are presented in Table 2. Approximately one-third of participants were aged between 25 and 29 years. Half of them were housewives and had completed secondary school. The majority were Muslim and were in poor economic status with monthly income lower than 156 USD. Underweight (BMI <18.5 kg/m^2^) was detected in 16.8% of the participants.

**Table 2:** Description of participant characteristics (N = 400)

Factor	n	%
Age (years)		
15-19	48	12.0
20-24	90	22.5
25-29	126	31.5
30-34	71	17.8
> 35	65	16.2
Occupation		
Housewife	193	48.3
Laborer	114	28.5
Other (agriculture/government employee/trader)	93	23.2
Religion		
Islam	377	94.2
Buddhism	23	5.8
Education achieved		
Primary school	165	41.2
Secondary school	190	47.6
Higher than secondary school	45	11.2
Gestational age at enrolment (weeks)		
12-14	134	33.5
15-17	139	34.7
17-20	127	31.8
Family income (baht)		
< 5,000	238	59.5
5,000-15,000	150	37.5
> 15,000	12	3.0
Number of family members		
1-5	246	61.5
6-10	144	36.0
11-15	10	2.5
Number of living children		
None	149	37.2
1-2	178	44.5
3-4	46	11.5
> 4	27	6.8
Body mass index (kg/m^2^)		
< 18.5	67	16.8
18.5 - 29.9	310	77.4
> =30.0	23	5.8
		

### Characteristics of nutrients intakes

Table 3 describes nutrient intake among the participants compared to the Thai RDA. Carbohydrate, protein, fat, calories, calcium, phosphorus, iron, thiamine, riboflavin, retinol, niacin, vitamin C, folic acid and iodine inadequacy was 86.8%, 59.2%, 78.0%, 83.5%, 55.0%, 29.5%, 45.2%, 85.0%, 19.2%, 3.8%, 43.2%, 0.8%, 0.0% and 0.8% respectively. The RDAs for all measured macronutrients, minerals and thiamine were in the fourth quartile of participant's nutrient intakes. The RDAs for retinol, vitamin C, folic acid and iodine were in the first quartile. If two-thirds of the RDA is considered as inadequate, the prevalence of inadequate macronutrient intake was over 59%.

**Table 3:** Description of daily nutrient intakes per day compared to RDA

Nutrient	RDA	Range	Mean intake	Quartile levels			Prevalence of inadequacy^a^ (%)		
					
				25%	50%	75%	< 50%	< 66.7%	< 80%
Macro-nutrients									
Total CHO (g)	345.0	51.7 - 416.8	177.6	136.2	178.3	214.2	47.2	86.8	95.5
Total protein (g)	58.0	6.8 - 100.5	37.1	28.2	36.5	44.0	27.0	59.2	81.5
Total fat (g)	75.0	1.8 - 94.0	38.9	27.9	34.5	47.6	57.2	78.0	87.0
Total calories (Kcal)	2,300	327.6 - 2724.5	1204.5	952.4	1208.6	1390.4	44.8	83.5	95.2
Micro-nutrients Minerals									
Calcium (mg)	800.0	50.7 - 1282.7	493.2	370.6	505.4	620.7	30.5	55.0	78.2
Phosphorus (mg)	700.0	107.5 - 1386.0	571.8	434.1	563.9	686.8	12.0	29.5	49.2
Iron (mg)	24.7	4.6 - 52.8	17.6	13.2	17.5	21.7	20.5	45.2	64.2
Vitamins									
Thiamine (mg)	1.4	0.1 - 2.5	0.7	0.5	0.7	0.7	51.7	85.0	95.8
Riboflavin (mg)	1.4	0.1 - 4.1	1.3	1.0	1.3	1.6	9.2	19.2	33.2
Retinol (μg RE)	800.0	80.1 - 13427.0	2366.5	1751.4	2096.3	3062.1	3.8	3.8	4.5
Niacin(mg)	18.0	4.1 - 32.8	9.8	12.8	15.2	32.8	18.2	43.2	67.8
Vitamin C (mg)	85.0	36.3 - 893.7	251.7	172.6	238.7	325.9	0.5	0.8	1.5
Folic acid (μg)	600.0	487.7 - 5411.4	1886.5	1406.28	1849.9	2293.3	0.0	0.0	0.0
Iodine (μg)	200.0	112.0 - 1101.0	379.9	294.3	374.1	450.1	0.0	0.8	2.0

RDA: Recommended Dietary Allowance for Thailand; CHO: Carbohydrate

^a^Inadequacy defined as nutrient intake for each cut point of Thai RDA

### Factors influencing inadequacy of nutrient intake

Due to the low prevalence of folic acid, vitamin C, iodine, retinol and riboflavin intake inadequacy in our sample demonstrated, these variables were excluded from the analysis of micronutrients. Tables 4 and 5 present the final model of logistic regression predicting inadequacy of macronutrients and micronutrients, respectively. Maternal age, educational level, gestational age at enrolment, pre-pregnancy BMI and level of violence were associated with nutrient inadequacies.

**Table 4:** Final logistic regression analysis on significant factors of macronutrient inadequacy with two-thirds of RDA levels^a^

	Macronutrients							
	Carbohydrate		Protein		Fat		Calorie	
	
	Adj. OR (95%CI)	p-value^b^	Adj. OR (95%CI)	p-value^b^	Adj. OR (95%CI)	p-value^b^	Adj. OR (95%CI)	p-value^b^
**Age **(years)		0.022		0.023		0.005		0.036
< 20	1.38 (0.49,3.89)		0.43 (0.22,0.83)*		0.40 (0.20,0.80)*		0.51 (0.24,1.12)	
20-34	1		1		1		1	
≥35	0.35 (0.16,0.75)**		0.64 (0.36,1.15)		0.41 (0.21,0.81)*		0.42 (0.20,0.87)*	
								
**Education**		0.042		< 0.001		< 0.001		< 0.001
Primary school	2.97 (1.11,7.91)*		2.23 (1.09,4.55)*		3.28 (1.37,7.89)**		3.51 (1.40,8.78)**	
Secondary school	1.38 (0.58,3.29)		0.94 (0.48,1.85)		0.19 (0.42,1.95)		1.16 (0.52,2.58)	
Higher than secondary school	1		1		1		1	
								
**Gestational age **(weeks)				0.023		0.034	-	
< 14		-	1		1			
≥14	-		0.46 (0.27,0.79)**		0.49 (0.25,0.98)*			
**Body mass index**		0.052	-		-		-	
< 18.5	0.45 (0.21,0.95)*							
18.5-29.9	1							
≥30.0	1.44 (0.62,3.36)							
								
**Weighted violence score**	1.66 (1.14,2.42)**	0.006	1.38 (1.08,1.77)**	0.009	-		-	

^a ^First model of logistic regression included age, education, religion, occupation, household income, gestational age at enrolment, number of family members and level of violence; ^b ^p-value by Likelihood Ratio Test; Adj. OR: Adjusted Odds Ratio; *p value < 0.05; ** p value <0.01

**Table 5:** Final logistic regression analysis on significant factors of micronutrient inadequacy with two-thirds of RDA levels^a^

	Micronutrients									
	Calcium		Phosphorus		Iron		Thiamine		Niacin	
	
	Adj. OR (95%CI)	p-value^b^	Adj. OR (95%CI)	p-value^b^	Adj. OR (95%CI)	p-value^b^	Adj. OR (95%CI)	p-value^b^	Adj. OR (95%CI)	p-value^b^
**Age **(years)					-		-			0.085
< 20	-		-						0.50 (0.25,0.98)*	
20-34		-		-					1	
≥35	-		-						0.70 (0.35,1.26)	
										
**Education**		0.001		0.004	-		-			0.085
Primary school	1.43 (0.73,2.83)		2.5 (1.12,5.6)*						2.71 (1.29,5.72)**	
Secondary school	0.64 (0.33,1.25)		1.25 (0.56,2.82)						1.59 (0.77,3.28)	
Higher than secondary school	1		1						1	
										
**Gestational age **(weeks)	-		-		-		-			0.002
< 14									1	
≥14									0.57 (0.35,0.95)*	
										
**Weighted violence score**	-		1.49 (1.16,1.92)**	0.002	1.63 (1.29,2.07)***	< 0.001	1.70 (1.20,2.41)**	0.002	1.80 (1.41,2.30)***	< 0.001

^a ^First model of logistic regression included age, education, religion, occupation, household income, gestational age at enrolment, number of family members and level of violence; ^b ^p-value by Likelihood Ratio Test; Adj. OR: Adjusted Odds Ratio

*p value < 0.05; ** p value <0.01; *** p value <0.001

Women aged less than 20 years were at a lowest risk of macronutrient and micronutrient inadequacy compared to those aged 20-34 years. Women with a primary school education were at a high risk for inadequacy of most nutrients. Gestational age was consistently associated with most nutrient inadequacies. If gestational age was more than 14 weeks, the risk of having macronutrient and micronutrient inadequacy was lower. Pre-pregnancy BMI was associated with only carbohydrate inadequacy. Women with low pre-pregnancy BMI had a lower risk of inadequacy compared to those with normal BMI. Level of violence, represented by weighted violence score in the area, was associated with a high risk of inadequacy for most macronutrients and micronutrients.

## Discussion

More than half of the women in this study were found to have nutrient intake inadequacy for all macronutrients plus calcium and thiamine. Almost half of the women had a lower iron and niacin intake compared to than the Thai RDA. Folic acid, vitamin C, iodine, retinol and riboflavin intakes were found to be adequate in most women. Factors associated with higher nutrient inadequacy were low educational attainment and high level of violence within the community. Those associated with lower nutrient inadequacy were maternal age less than 20 years, gestational age at enrolment of 14 weeks or more and a pre-pregnancy BMI of less than 18.5.

Table 6 shows a summary of five studies conducted in the USA, Nigeria, China and Thailand on the levels of daily nutrient intake in pregnant women [4,6,7,10,11]. The gestational age at recruitment and definition of inadequate intake varied across studies. In our study, gestational age and definition of inadequacy was similar to the study from USA [7] but different from the other two studies in Thailand, which both studied pregnant women in their late trimester [10,11]. Inadequacy of both macronutrient and micronutrient intake was commonly detected in developing countries [4,6] in contrast to the study from USA [7].

**Table 6:** A summary of daily nutrient intake among pregnant women

Variables	This study		**Piammongkol et al., 2004 **[10]		**Jaruratanasirikul et al., 2009 **[11]		**Cheng et al., 2009 **[4]		**Oguntona and Akinyele, 2002 **[6]		**Swensen et al., 2001 **[7]	
**Site**	**Hospital-based Narathiwat, southern Thailand**		**Community-based Pattani, southern Thailand**		**Hospital-based Songkhla, southern Thailand**		**Hospital-based Shaanxi, western China**		**Hospital-based Ogun state, Nigeria**		**Hospital-based Mineapolis & St. Paul, USA**	

**Gestational age**	**12-20 weeks**		**32-40 weeks**		**12-16 weeks**		**< 24 weeks**		**< 24 weeks**		**< 20week**	
**Recommended considered**	**2/3 (66.7%)RDA**		**80%RDA**		**100% iDRI**		**100% DRI**		**100%RDA**		**2/3 (66.7%)RDA**	

**Nutrient intake**	**% of inadequacy**	**Mean**	**% of inadequacy**	**Mean**	**% of inadequacy**	**Mean**	**% of inadequacy**	**Mean**	**% of inadequacy**	**Mean**	**% of inadequacy**	**Mean**
Macronutrient												
- CHO(g)	86.8	177.6	-	227.1		225.0	-	391.2	-	263.5		
- Protein(g)	81.5	37.1	46.4	48.9	-	70.0	31.0	63.4	44.0	39.8	2.0	-
- Fat(g)	87.0	38.9	-	20.2	-	60.0	-	57.0	-	23.6	-	-
- Calories(Kcal)	95.2	1,204.5	96.4	1,285.0	-	1,806.0	54.0	2,331.9	44.3	1,405.8	30.0	-
Micronutrient												
- Thiamine(mg)	85.0	0.7	98.8	0.4	-	0.46	-	1.4	-	-	5.0	-
- Riboflavin(mg)	19.2	1.3	75.3	0.9	-	1.33	91.0	0.9	-	-	0.0	-
- Niacin(mg)	43.2	9.8	29.5	16.2	-	17.0	-	12.2	-	-	12.0	-
- Vitamin A(ng RE)	3.8	2,366.5	3.6	2,628.0	-	718.0	-	572.0	0.0	2,387.0	3.0	-
- Vitamin C(mg)	0.8	251.7	14.5	119.. 0	-	71.0	34	106.3	-	-	8.0	-
- Calcium(mg)	55.0	493.2	99.4	281.7	-	700.0	70.0	453.7	25.1	748.8	17.0	-
- Phosphorus(mg)	29.5	571.8	97.6	431.3	-	698.0	-	1,147.6	-	-	0.0	-
- Iron(mg)	45.2	17.6	98.8	22.0	-	16.0	-	23.2	62.0	11.4	90.0	-
- Iodine(μg)	0.8	379.9	-	-	-	-	-	-	-	-	-	-
- Zinc(mg)	-	-	-	-	-	3.5	91.0	8.9	18.7	12.2	24.0	-
- Copper(mg)	-	-	-	-	-	-	-	1.9	73.3	0.8	-	-
- Folate(μg)	-	-	-	-	-	-	97.0	265.9	67.0	198.0	-	-

"-" No information available; RDA: Recommended Dietary Allowance; DRI: Dietary Reference Intakes

Our study found that maternal age, education, gestational age at recruitment and pre-pregnancy BMI were associated with nutrient inadequacy. Maternal age was associated with inadequacy of all macronutrients. In our study, pregnant women aged less than 20 years, compared to those aged 20-34 years, had a lower nutrient inadequacy. This finding was different from the study in Nigeria which showed a higher inadequacy and 100% RDA was used for the threshold of inadequacy [6]. This finding discrepancy may be explained by the different threshold of inadequacy and difference in study participants. Pregnancy among youths in lower south of is socially acceptable; as a result, young women may have awareness on the risks of pregnancy leading to improved nutrient intakes.

Education was found to have a consistent effect on inadequacy of almost all nutrients. Women who had a primary education or less were 1.5-3 times more likely to have nutrient inadequacy. Generally, nutritional principles are incorporated into the compulsory education of Thailand. However, knowledge on nutrition requirement for pregnant women is not included. It is also possible that women with a higher education have better food habits than those with a low education. This is supported by a study in Japan which found that higher education is associated with higher intakes of protein, fatty acid, dietary fiber, cholesterol, potassium, calcium, magnesium, iron, vitamin A, D, E and C and folate [13].

In addition, gestational age at enrolment of 14 weeks or more was found to be associated with a lower risk for inadequacy of both macronutrients and micronutrients. This finding was consistent with a previous study which found that a loss of appetite and morning sickness were more likely to occur in the first trimester of pregnancy [19]. Women with pre-pregnancy BMI of less than 18.5, compared to normal BMI (BMI 18.5-24.9), had a lower carbohydrate inadequacy in our study, contrasting a finding of a study in China [4]. This might be explained by a difference in definition of inadequacy between two studies. However, the link between lower carbohydrate inadequacy and low pre-pregnancy BMI could not be explained. Although a study suggested that the type of carbohydrate may be related to body weight, the daily total carbohydrate intake was not found to be related to BMI [20].

A higher level of violence in the community was associated with an increased risk of all nutrients inadequacy except fat, calorie and calcium. This finding might be due to the agricultural nature and lifestyles of people in this area. It is quite common for people living in the study area to set aside land surrounding their house for gardening and farming. Locally grown fruits and vegetables are rich in nutrients and provide the residents with adequate sources of calcium. Fresh fruits, such as banana, durian, jackfruit, rambutan and coconuts, which are eaten throughout southern Thailand, contain high amounts of fat and calories. The effect of long-term violence on nutrition intake may result in increased stress [21,22], which may subsequently decrease appetite [23,24]. In addition, foods may also be less accessible in areas of violence due to safety problems and difficulties involved with food transportation. These and other consequences of violence eventually reduce nutrient uptake for the people living in the area leading to nutrient inadequacy.

This study was hospital-based in nature but the coverage of early antenatal care was high (90%). Therefore the problem of samples not being representative of the population may not be serious. Like most epidemiological nutritional surveys, the reliability of food intake based on tabulation may be doubtful. Assuming that recall errors occur at random, the results would be biased toward no association.

## Conclusions

Pregnant women living in Narathiwat province of southern Thailand had a high prevalence of nutrient intake inadequacy, particularly in macronutrients. Maternal age, education, gestational age, pre-pregnancy BMI and level of violence were associated with nutrient intake inadequacy; however, all the dietary intake components were not equally affected. Our evidence suggests that both individual and community factors are important and there is a need to put more effort on good counseling at antenatal care clinics to alleviate nutrient inadequacy, especially in high-risk women. The strengthening of community, health providers and health systems roles are needed for further studies to improve this condition.

## Competing interests

The authors declare that they have no competing interests.

## Authors' contributions

PS, TL, VC and PS designed the study, coordinated the data collection, analyzed and interpreted the data and prepared the manuscript. VS and MK contributed to the data management of the violence data and edited the manuscript. All authors read and approved the final manuscript.

## Pre-publication history

The pre-publication history for this paper can be accessed here:

http://www.biomedcentral.com/1471-2458/10/572/prepub
